# A Changing Number of Alternative States in the Boreal Biome: Reproducibility Risks of Replacing Remote Sensing Products

**DOI:** 10.1371/journal.pone.0143014

**Published:** 2015-11-16

**Authors:** Chi Xu, Milena Holmgren, Egbert H. Van Nes, Marina Hirota, F. Stuart Chapin, Marten Scheffer

**Affiliations:** 1 School of Life Sciences, Nanjing University, Xianlin Road 163, Nanjing, 210023, P.R. China; 2 Aquatic Ecology and Water Quality Management Group, Wageningen University, P.O. Box 47, NL-6700 AA, Wageningen, The Netherlands; 3 Resource Ecology Group, Wageningen University, P.O. Box 47, NL-6700 AA, Wageningen, The Netherlands; 4 Department of Physics, Federal University of Santa Catarina, P.O. Box 476, 88040–970, Florianópolis, Brazil; 5 Institute of Arctic Biology, University of Alaska, Fairbanks, Alaska, 99775, United States of America; Center for International Forestry Research (CIFOR), INDONESIA

## Abstract

Publicly available remote sensing products have boosted science in many ways. The openness of these data sources suggests high reproducibility. However, as we show here, results may be specific to versions of the data products that can become unavailable as new versions are posted. We focus on remotely-sensed tree cover. Recent studies have used this public resource to detect multi-modality in tree cover in the tropical and boreal biomes. Such patterns suggest alternative stable states separated by critical tipping points. This has important implications for the potential response of these ecosystems to global climate change. For the boreal region, four distinct ecosystem states (i.e., treeless, sparse and dense woodland, and boreal forest) were previously identified by using the Collection 3 data of MODIS Vegetation Continuous Fields (VCF). Since then, the MODIS VCF product has been updated to Collection 5; and a Landsat VCF product of global tree cover at a fine spatial resolution of 30 meters has been developed. Here we compare these different remote-sensing products of tree cover to show that identification of alternative stable states in the boreal biome partly depends on the data source used. The updated MODIS data and the newer Landsat data consistently demonstrate three distinct modes around similar tree-cover values. Our analysis suggests that the boreal region has three modes: one sparsely vegetated state (treeless), one distinct ‘savanna-like’ state and one forest state, which could be alternative stable states. Our analysis illustrates that qualitative outcomes of studies may change fundamentally as new versions of remote sensing products are used. Scientific reproducibility thus requires that old versions remain publicly available.

## Introduction

A recent call for more transparent, open and reproducible science stressed the need to have data posted in a trusted repository [1]. Numerous scientific studies are now based on publicly available remote-sensing products that seem to fit this criterion well. However, as we show here, there is a serious reproducibility issue if old versions of products become unavailable.

Recent studies have used remotely-sensed and field-measured tree cover to detect multi-modality in the tree cover frequency distribution [2–5]. If the environmental conditions such as rainfall and soil characteristics have a unimodal frequency distribution, marked modes in the frequency distribution of the state of a system may indicate the existence of alternative stable states [6]. In the case of tree cover this interpretation has important implications for understanding ecosystem responses to global climate change. Some potential caveats of interpreting remotely-sensed tree cover have been pointed out [7–9]. Here we address the fundamental issue that results may substantially change with the version of the product used. This precludes reproduction of the findings if the old version is no longer available.

For the boreal region, Scheffer et al. [3] inferred the existence of four distinct states by quantifying the modes of frequency distribution of tree cover, using the Collection 3 data of MODIS Vegetation Continuous Fields (VCF) [10]. Since then, the MODIS VCF product has been updated to Collection 5 [11]; and a Landsat VCF product of global tree cover at a fine spatial resolution of 30 meters has been developed [12]. Here we show that identification of alternative stable states in the boreal biome partly depends on the data source used, by comparing the results from these different remote-sensing products of tree cover.

## Materials and Methods

There are major differences between these two versions of MODIS VCF data. According to the authors, new high-resolution training data and the implementation of improved data mining software have resulted in much greater accuracy in the new Collection 5 product [13]. To assess the consequence of such differences, we used the same sampling points (locations and sample dates) in the boreal region and processed the data in the same way as in our previous work [3].

## Results and Discussion

**Fig 1:**
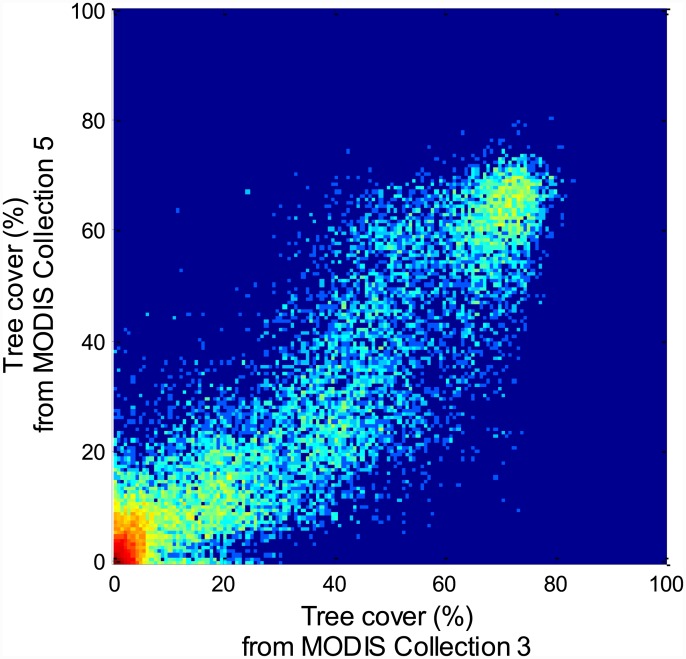
Scatter density plot of tree cover in 2001 from MODIS Collection 3 against Collection 5. Point density from low to high is indicated by the color gradient from blue to red.

**Fig 2:**
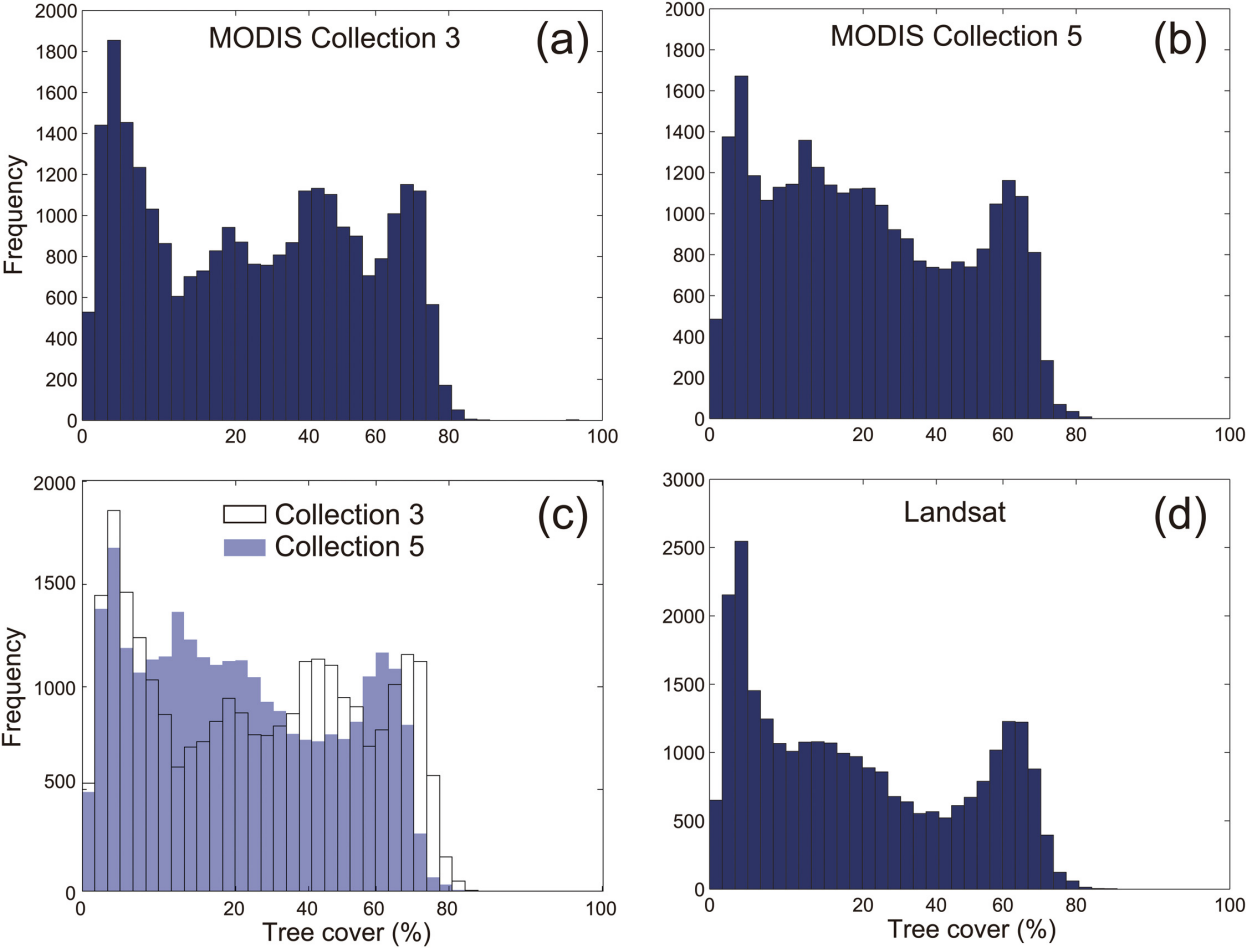
Comparison of tree-cover frequency distribution from different data sources. MODIS Collection 3 (a) and Collection 5 (b) in 2001, frequency difference between these two versions at each tree-cover bin (c) and Landsat VCF data (d) in 2000. Tree cover percentage values have been transformed through the arcsine-squared-root transformation.

The strong correlation (Pearson's *r* = 0.861, *P* < 0.001, *n* = 29 052) implies general consistency between the Collections 3 and 5 (Fig 1). However, ~35% of the sampling points differ by >10% of tree cover between Collections 3 and 5. Importantly, the frequency distribution of tree cover, used as the basis for inferring multi-modality in tree cover, shows substantial discrepancy. Four modes were previously identified from the Collection 3 product, representing boreal forest (∼75% tree cover), dense and sparse woodland (∼45% and ∼20% tree cover respectively), and a treeless state (Fig 2a). The new version of the MODIS VCF product still shows a clear multi-modal frequency distribution of tree cover. However, the modes representing sparse woodland and boreal forest now occur at a ~10% lower tree cover (Fig 2b), and the separate mode for dense woodland is not statistically significant anymore (tested by using latent class analysis performed in the *gmdistribution* function of MATLAB, S1 Fig). A closer look reveals that the Collection 3 product generally underestimates tree cover around 10–30% and 60%, while it overestimates tree cover around 40% and 70% (Fig 2c).

A mechanistic explanation of these differences would require analysis of the inherently complex calibration procedures of the VCF product [9]. However, the tri-modality suggested by the newer data seems a more robust result than the previously reported result with two (dense and sparse) woodland modes. Tri-modality is consistently detected at multiple temporal and spatial scales. During 2000–2010, annual tree cover from the MODIS Collection 5 repeatedly shows three distinct modes, where the two modes representing boreal forest (~65% tree cover) and treeless state (~5% tree cover) remain highly stable; and one mode (between 15–25% tree cover) representing ‘savanna-like’ woodland is statistically significant across the 11 years (S2 Fig). Note that our analysis on these multi-temporal data aims at checking consistency of frequency distribution, rather than comparing temporal system dynamics (see [14] for the caveat of inter-annual comparison of MODIS VCF data). Being well in line with the 250-m MODIS VCF data, the 30-m resolution Landsat VCF data also detects three distinct modes around similar tree-cover values than MODIS Collection 5 (Fig 2d). Validation work has demonstrated that the Landsat VCF data with finer resolution have higher accuracy than the MODIS VCF data [12]. Particularly, the Landsat data show substantially improved performance for estimating sparse tree covers, where the MODIS data have been criticized to have greater errors [7, 15], mostly due to the limitation of spatial resolution. The consistent results from these two different remote-sensing products provide more robust evidence of tri-modality pattern of tree cover in the boreal region.

## Conclusions

Our new analysis thus suggests that, much as in the tropics [2, 16], the boreal region has three modes: one sparsely vegetated state, one distinct ‘savanna-like’ state and one forest state, which could be alternative stable states. This could reflect sensitivity to similar processes (e.g., disturbance) between the two biomes and suggests that both biomes might undergo rapid and potentially irreversible biome shifts if climate change alters the processes responsible for multi-modal patterns [2]. These results illustrate how profoundly results may change depending on the version of a remote sensing product used. Clearly, new versions of products do usually imply an improvement. However, our findings illustrate that it is important to keep old versions available to allow independent reproduction of the results.

## Supporting Information

S1 Fig Comparison of fitting 1–5 normal distributions to tree cover based on the Bayesian Information Criterion (BIC). (PDF)

S2 Fig Frequency distribution of annual tree cover from MODIS Collection 5 during 2000–2010. (PDF)
