# Supplementary material for: A Changing Number of Alternative States in the Boreal Biome: Reproducibility Risks of Replacing Remote Sensing Products
Source: PLoS One. 2015 Nov 16;10(11):e0143014. doi: 10.1371/journal.pone.0143014 (PMC4646617; doi:10.1371/journal.pone.0143014)

S1. Fig. Comparison of fitting 1-5 normal distributions to tree cover based on the Bayesian Information Criterion (BIC). The model with the minimal BIC (i.e., 3 distributions) shows the optimum fit (a), and the fitted probability density function curve (b). Before analysis, tree cover was arcsine-square-root transformed and a random subsample of 1000 points was taken.

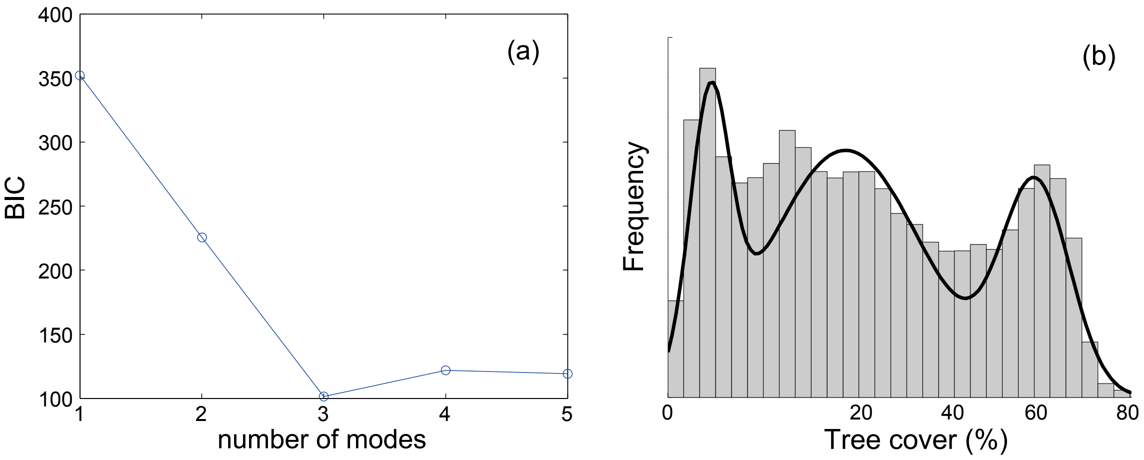

Supplement: S1 Fig — (PDF) [file pone.0143014.s001.pdf]
