# Supplementary material for: A Changing Number of Alternative States in the Boreal Biome: Reproducibility Risks of Replacing Remote Sensing Products
Source: PLoS One. 2015 Nov 16;10(11):e0143014. doi: 10.1371/journal.pone.0143014 (PMC4646617; doi:10.1371/journal.pone.0143014)

S2. Fig. Frequency distribution of annual tree cover from MODIS Collection 5 during 2000-2010. Tree cover percentage values have been transformed through the arcsine-squared-root transformation.

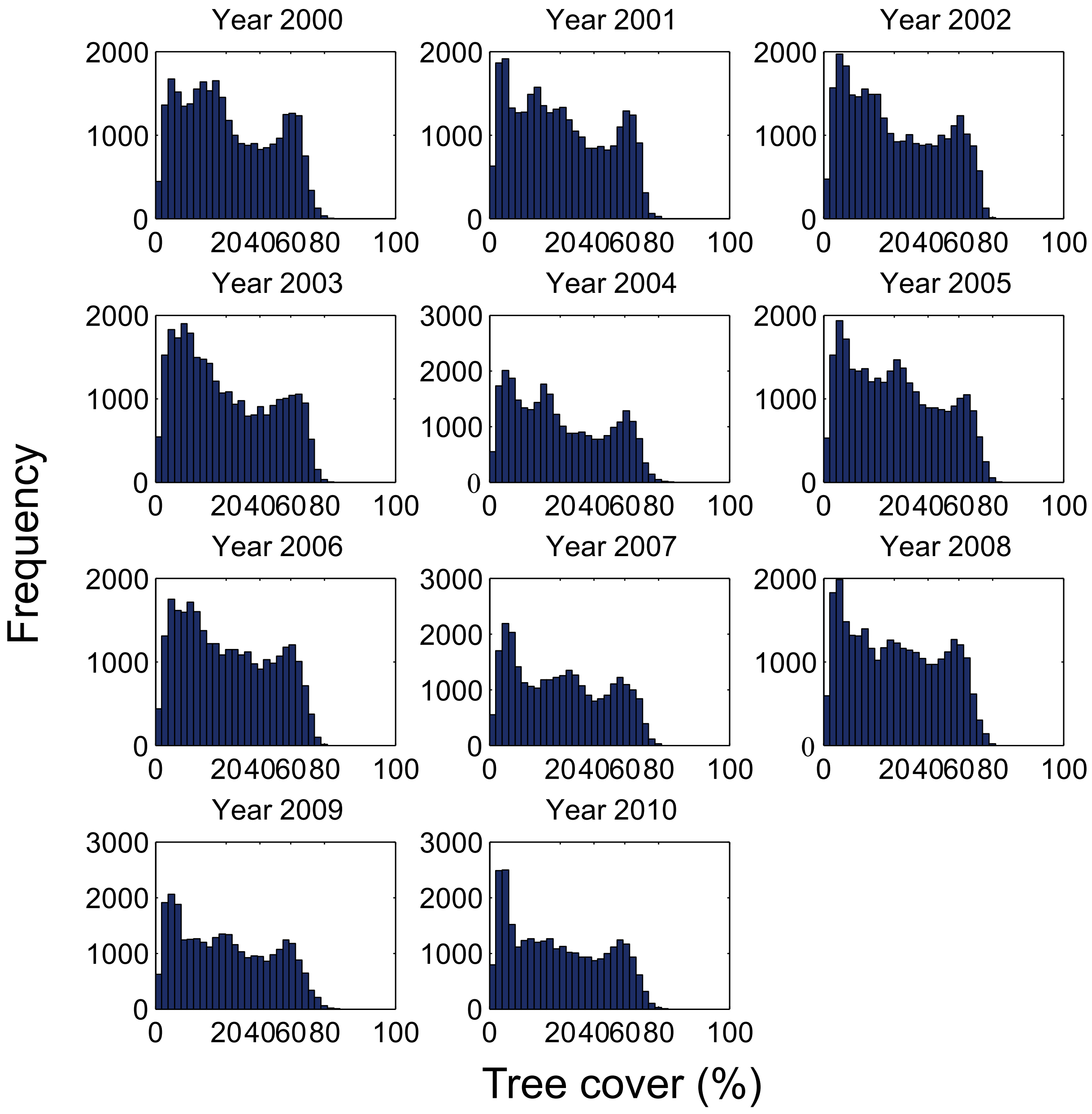

Supplement: S2 Fig — (PDF) [file pone.0143014.s002.pdf]
